# Partial portal vein arterialization during living-donor liver transplantation: a case report

**DOI:** 10.1186/s40792-020-0781-8

**Published:** 2020-01-08

**Authors:** Yasuhiro Maruya, Masaaki Hidaka, Florian Pecquenard, Alzhan Baubekov, Yuki Nunoshita, Shinichiro Ono, Tomohiko Adachi, Mitsuhisa Takatsuki, Katsumi Tanaka, Shinichiro Ito, Kengo Kanetaka, Susumu Eguchi

**Affiliations:** 1 Department of Surgery, Graduate School of Biomedical Sciences, Nagasaki University, 1-7-1 Sakamoto, Nagasaki, 852-8501 Japan; 2 Department of Plastic and Reconstructive Surgery, Graduate School of Biomedical Sciences, Nagasaki University, Nagasaki, Japan; 3 Department of Surgery, A.N. Syzganov’s National Scientific Center of Surgery, Almaty, Kazakhstan

**Keywords:** Partial portal vein arterialization, Living-donor liver transplantation

## Abstract

**Background:**

Hepatic artery thrombosis can lead to graft loss associated with severe hepatic infarction or bile duct ischemia. When anatomical hepatic artery reconstruction is impossible in liver transplantation or hepato-pancreatic biliary surgery, portal vein arterialization (PVA) is proposed as a salvage technique. Herein, we report our experience with a case that showed favorable clinical outcomes after partial PVA during living-donor liver transplantation (LDLT) because of difficulties in arterial reconstruction.

**Case presentation:**

A 62-year-old woman with non-B, non-C liver cirrhosis complicated with hepatocellular carcinoma was being prepared for LDLT using an extended left lobe graft. The graft presented with two arteries (left hepatic artery, 2 mm; middle hepatic artery, 2 mm). The first anastomosis was performed using the recipient hepatic artery stumps, but no flow was detected on Doppler control because of thrombus formation. The next attempt was executed using the middle colic artery with a radial artery jump graft and the right gastroepiploic artery, but it led to the same result. Thus, the graft oxygen support by the standard arterial procurement was abandoned, and a shunt was created between the ileocecal artery and the vein to obtain PVA. Arteriography of the superior mesenteric artery showed that the shunt was relatively patent, and the portal vein was apparent. No biliary complication or liver abscess occurred postoperatively, and the patient presented with good liver function and no complications related to portal vein hypertension, nor liver fibrosis 18 months after the LDLT.

**Conclusion:**

Partial PVA with a shunt created between the ileocecal artery and the vein is useful when arterial reconstruction is difficult during LDLT for preventing graft loss caused by severe hepatic infarction or bile duct ischemia.

## Introduction

In liver transplantation, restoration of oxygenated blood supply to the liver allograft is necessary, considering that all collateral sources of arterial inflow (interlobar hepatic arteries, peribiliary plexus, and collaterals in triangular ligaments and lesser omentum) are completely interrupted during graft harvest [1]. Thus, when anatomical hepatic artery reconstruction is impossible, a totally de-arterialized liver may occur. If the hepatic artery is not reconstructed, biliary ischemia and necrosis can occur, leading to liver necrosis with fatal liver failure. Re-transplantation is the standard procedure when anatomical hepatic artery reconstruction is difficult; however, re-transplantation is not always possible because of donor shortage in a country such as Japan.

Nonetheless, portal vein arterialization (PVA) has been used as a salvage technique in case of difficulties in anatomical hepatic artery reconstruction during liver transplantation and hepato-pancreatic biliary (HPB) surgery [1–3]. PVA increases the oxygen saturation of portal vein blood significantly, preventing hepatic necrosis and failure and promoting liver regeneration [1, 4–6]. It also avoids biliary ischemia through terminal portal vein tributaries anastomosed with the arterial peribiliary plexus [1, 6].

Herein, we report our experience with a case in which partial PVA was useful for difficult arterial reconstruction during a living-donor liver transplantation (LDLT).

## Case report

**Fig. 1:**
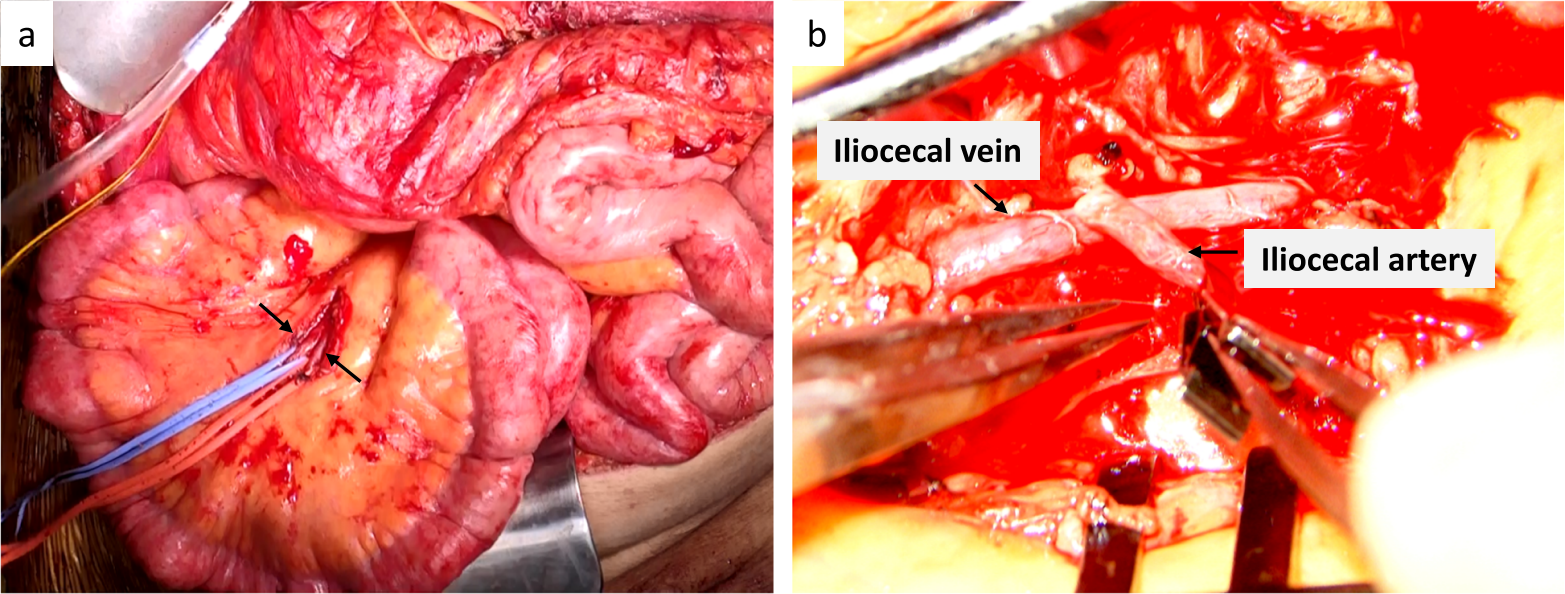
**a** Intraoperative image of an opened mesoileum and selected ileocecal artery and vein, which were taped (arrow). **b** Ileocecal artery anastomosed to the vein in an end-to-side fashion with 9-0 Nylon

A 62-year-old Japanese female was referred to Nagasaki University Hospital to discuss an LDLT for recurrent hepatocellular carcinoma (HCC) and end-stage liver disease due to non-B, non-C liver cirrhosis. Two years earlier, she underwent trans-arterial chemoembolization. During follow-up, her liver function gradually worsened with refractory ascites. She then repeatedly received a cell-free and concentrated ascites reinfusion therapy for severe ascites. A new HCC lesion with a 1.5-cm nodule located in segment 7 was also detected by enhanced computed tomography (CT). The Child–Pugh score was 9 (grade B), and the Model for End-stage Liver Disease score was 16. Her dismal status indicated LDLT, and after an appropriate workup, an extended left lobe graft from her 35-year-old healthy daughter was voluntarily donated. Preoperative imaging findings of the donor did not exhibit calcification of the arteries or anatomical variation. Potential causes of failure of hepatic arterial reconstruction, such as a median arcuate ligament syndrome, were not present, and potential vessel injuries by previous trans-arterial chemoembolization had not occurred in the recipient. Donor surgery was performed without any difficulty in the procurement of the extended left lobe graft. The graft’s two hepatic arteries—the left hepatic artery (LHA) and the middle hepatic artery (MHA)—were anastomosed with the recipient’s counterpart microscopically, but it led to thrombosis at numerous attempts. After confirming blood flow in the anastomosed hepatic artery, we performed duct-to-duct biliary reconstruction. We noticed afterwards that we could not confirm blood flow in the hepatic artery with the Doppler US probe. Anastomosis using the recipient’s middle colic artery to the graft’s LHA microscopically through a right radial artery jump graft was attempted with no arterial flow on Doppler control. Thereafter, the arterial oxygen supply was abandoned, and an arteriovenous shunt was created between the selected ileocecal artery and the vein to obtain PVA. We used mesenteric blood vessels to perform selective portal arterialization, in reference to Hayashi et al.’s report [2]. Because the operative field of this procedure was separated from the hepatic hilum, it was easy to approach not only when we created the partial portal arterialization but also when we closed the shunt to prevent symptoms of portal hypertension, after confirmation of collateral arterial blood flow [2]. The mesoileum was opened, and the selected ileocecal artery and vein were taped (Fig. 1a). The ileocecal artery was anastomosed to the vein microscopically in an end-to-side fashion with 9-0 Nylon (Fig. 1b). After anastomosis, ultrasonography confirmed favorable blood flow in the portal and hepatic veins. Portal vein pressure was not measured, and we did not perform any modulation intraoperatively. The operative time was 1737 min, and the blood loss was 23,314 g. The time from portal reperfusion to recovery of arterial oxygenation was 1240 min.

**Fig. 2:**
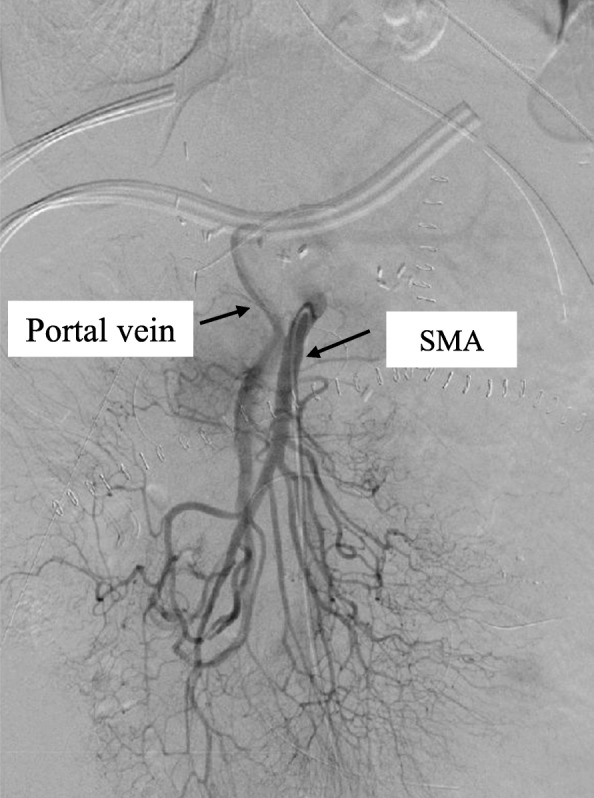
Angiography of the superior mesenteric artery was performed on POD 3, showing a patent shunt and a clearly visible portal vein (arrow)

**Fig. 3:**
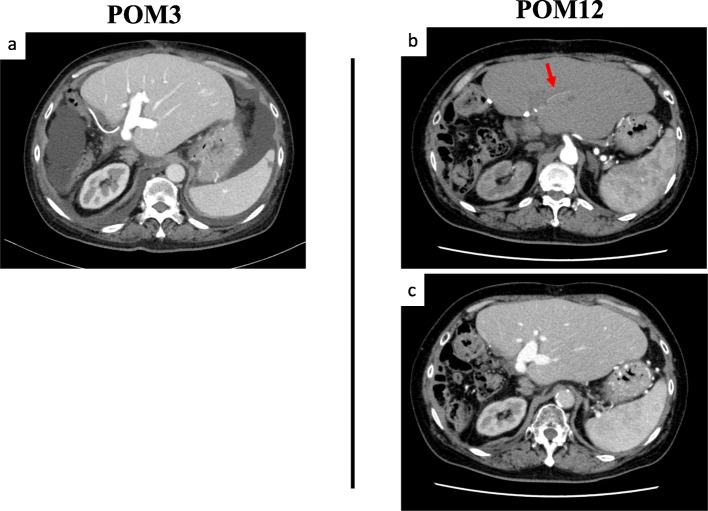
**a** CT scan performed on POM 3 showing that the portal vein was enhanced through the ileal arteriovenous shunt at the arterial phase. **b** CT scan performed on POM 12 showing that a thin hepatic artery (arrow), which indicated improved arterialization through bile duct anastomosis, was visible during the early arterial contrast phase. **c** The portal vein was enhanced through the ileal arteriovenous shunt on the late arterial phase

Laboratory analysis revealed that during the period of no oxygenation after portal reperfusion, the AST level was 100 U/L and the ALT level was 118 U/L; on postoperative day (POD) 1, these levels were 118 U/L and 89 U/L, respectively; on POD 2, 72 U/L and 149 U/L, respectively; on POD 3, 71 U/L and 145 U/L, respectively; on POD 4, 108 U/L and 159 U/L, respectively; and on POD 4, 78 U/L and 134 U/L, respectively. After POD 5, both levels decreased gradually. The patient’s serum total bilirubin levels were elevated on POD 1 but decreased gradually after the LDLT. Angiography of the superior mesenteric artery was performed on POD 3, showing a patent shunt and a clearly visible portal vein (Fig. 2). After transplantation, continuous hemodiafiltration was needed until POD 10 because of preoperative hepatorenal syndrome. On POD 13, this was switched to hemodialysis, which was withdrawn on POD 21. The amount of ascites was not severe after LDLT: approximately 2000 mL on POD 7 and approximately 300 mL on POD 14. We removed the drainage tube completely on POD 23. We checked portal flow with the Doppler US probe twice a day for 1 week after LDLT; however, we could not confirm an arterial waveform in portal flow, and in fact, we had never seen high flow volume of the portal vein on US study. Thereafter, the patient’s postoperative course was uneventful. On POD 67, she was discharged. During postoperative month (POM) 3, a CT scan was performed, showing that the portal vein was enhanced through the ileal arteriovenous shunt at the arterial phase (Fig. 3a). Then, during POM 12, another CT scan was performed, revealing that a thin hepatic artery, which might indicate improved arterialization through biliary anastomosis or via intrahepatic biliary plexus, was visible during the early arterial contrast phase (Fig. 3b). On the late arterial phase, the portal vein was enhanced through the ileal arteriovenous shunt (Fig. 3c). We measured the patient’s spleen volume with a three-dimensional image analysis system (SYNAPSE VINCENT; Fujifilm Corporation, Tokyo, Japan) before surgery, on POD 7, and during POM 1, POM 3, and POM 12. The spleen volume was 292 mL preoperatively, 386 mL on POD 7, 337 mL during POM 1, 339 mL during POM 3, and 261 mL during POM 12. The patient’s spleen volume was elevated on POD 7 but decreased gradually after the LDLT. During POM 12, we confirmed, with upper gastrointestinal endoscopy, that the esophageal varices had not progressed. No signs of portal hypertension, progression of esophageal varices, hepatic abscess, biliary complication, or fibrosis were noted after 18 months of follow-up. One year after the LDLT, no ischemic change of the bile duct, biliary necrosis, inflammation around the portal tract, or acute cellular rejection was detected on liver biopsy.

## Discussion

The liver receives dual blood supply from the portal vein and the hepatic artery. However, if the hepatic artery is interrupted, the blood flow and oxygen supply decrease, causing biliary ischemia, liver necrosis, and fatal liver failure. Nevertheless, ischemic cholangitis and bile duct necrosis do not occur in all patients with a de-arterialized liver because of the heterogeneous rapid development of collaterals after complete interruption of the hepatic artery in general and HPB surgery. Considering the complete interruption of all collateral sources of arterial inflow (interlobar hepatic arteries, peribiliary plexus, and collaterals in triangular ligaments and lesser omentum) during liver transplantation, oxygenated blood supply to the liver allograft by the hepatic artery is necessary. The hepatic artery plays a vital role after liver transplantation, especially after LDLT, providing blood for both the liver parenchyma and bile duct. Thus, if anatomical hepatic artery reconstruction is impossible during liver transplantation, a totally de-arterialized liver may occur. Eventually, biliary ischemia and necrosis take place, thereby possibly leading to liver necrosis and fatal liver failure. PVA, which allows arterial blood to flow into the portal vein, is an emergency salvage surgical procedure for cases wherein reconstructing the hepatic artery is difficult during liver transplantation and HPB surgery.

Iseki et al. studied human PVA in a de-arterialized liver of a patient in whom hepatic artery ligation following arterial rupture and hemorrhage after Whipple procedure was required [4]. Subsequently, the use of PVA has been reported in liver transplantation and in HPB surgery primarily for hepatic artery thrombosis (HAT) [6–10]. PVA is temporarily effective in preventing premature graft failure. However, the incidence rate of portal hypertension is approximately 44%, and it is more common within 1 year, including the portal arterialization cases of postoperative HAT [1]. Strict blood pressure control and shunt blood flow closure are needed, and careful consideration should be applied before selecting this modality of liver oxygenated blood supply restoration [1, 10].

According to some authors, calibrating the arterial inflow is important for preventing portal hypertension after PV [1, 9, 11]. Hayashi et al. reported that the portal pressure after constructing a partial PVA with a mesenteric arteriovenous shunt could be slight because the arterial flow into the portal vein is not excessive [2]. In our case, the patient presented with good liver function tests and no complications related to portal vein hypertension, nor liver fibrosis on POM 18. The reasons why complications related to portal vein hypertension did not occur in our case might be the construction of a partial PVA using a mesenteric arteriovenous shunt and the buffering effect of a liver graft from a young donor. However, if such complications shall occur, shunt closure or embolization might need to be considered.

## Conclusion

In conclusion, PVA with a shunt created between the ileocecal artery and the vein is useful in case of difficult arterial reconstruction during LDLT for preventing graft loss caused by severe hepatic infarction or bile duct ischemia.

## Abbreviations

CT: Computed tomography
HAT: Hepatic artery thrombosis
HCC: Hepatocellular carcinoma
HPB: Hepato-pancreatic biliary
LDLT: Living-donor liver transplantation
LHA: Left hepatic artery
MHA: Middle hepatic artery
POD: Postoperative day
POM: Postoperative month
PVA: Portal vein arterialization
SMA: Superior mesenteric artery
